# Metabolomics for Secondary Metabolite Research

**DOI:** 10.3390/metabo3041076

**Published:** 2013-11-11

**Authors:** Rainer Breitling, Ana Ceniceros, Andris Jankevics, Eriko Takano

**Affiliations:** 1 Manchester Institute of Biotechnology, University of Manchester, 131 Princess Street, Manchester M1 7DN, UK; E-Mails: rainer.breitling@manchester.ac.uk (R.B.); andris.jankevics@manchester.ac.uk (A.J.); 2 Microbial Physiology, Groningen Biomolecular Sciences and Biotechnology Institute, University of Groningen, Nijenborg 7, Groningen 9747 AG, The Netherlands; E-Mail: a.ceniceros@rug.nl

**Keywords:** metabolomics, secondary metabolites, mass spectrometry

## Abstract

Metabolomics, the global characterization of metabolite profiles, is becoming an increasingly powerful tool for research on secondary metabolite discovery and production. In this review we discuss examples of recent technological advances and biological applications of metabolomics in the search for chemical novelty and the engineered production of bioactive secondary metabolites.

## 1. Introduction

Secondary metabolites are small biomolecules considered to be non-essential for the life of the producer organism [1]. They provide the producer organism with survival advantages in various ways, for instance by improving nutrient availability (e.g., in the form of chelating agents such as siderophores), by protecting against environmental stressors (e.g., pigments and osmoprotectants), by enhancing competitive interactions with other organisms (e.g., antibiotics, but also various signalling molecules), or by acting as a metabolic defence mechanism (e.g., many plant flavonoid and alkaloid toxins).

Many secondary metabolites have great importance for humans. They are widely used as active drug ingredients in medicine (e.g., many antibiotics, antitumor agents and antivirals are derived from secondary metabolites, as are antipyretics like aspirin, hallucinogenics like LSD, and cholesterol-lowering drugs like lovastatin [2,3,4], as herbicides or phytotoxins in agriculture [5], as food additives (colour, flavours and sweeteners) [6], fragrances, and even as precursors for the synthesis of plastics [7].

The rapid development of genomics in the last years has helped us reveal that many organisms encode the potential to produce many more secondary metabolites than was originally expected. Most of these new secondary metabolites are only predicted by bioinformatics analysis of putative secondary metabolite gene clusters in sequenced genomes, but are not produced naturally under laboratory conditions or are present at levels that are too low to be detected by standard methods. In some cases, the production of such cryptic or sleeping secondary metabolites has been successfully induced by genetic manipulations [8,9]. The emerging methods of Synthetic Biology have recently resulted in renewed interest in the discovery of novel bioactive secondary metabolites from a wide variety of sources [10,11,12].

Metabolomics is a key component of the Synthetic Biology approach to secondary metabolite biology. It aims at discovering and characterizing secondary metabolites in their metabolic context in natural or engineered biosystems, by simultaneously measuring as many low molecular weight compounds as possible. Comprehensive and detailed overviews of metabolomics methods applied to plant studies, Synthetic Biology and pathogens have been presented recently [13,14,15,16,17,18]. The processing and interpretation of the large amounts of recorded data are particular challenges in metabolomics, and have also been reviewed in detail in several publications [19,20,21,22].

In the present review we discuss the power of metabolomics as a tool in the new generation of bioprospecting efforts targeting secondary metabolites.

## 2. Metabolomics for Secondary Metabolite Discovery

The most obvious application of metabolomics in secondary metabolites studies is as an analytical tool for rapidly detecting, characterizing and identifying compounds produced by an organism, especially with relatively limited knowledge of the chemical nature of the target. For example, Kersten *et al*. [23] found DNA-interfering activity in *Salinispora tropica* CNB-440 chemical extracts. Genome mining to identify the responsible genes detected two enediyene biosynthesis clusters in this strain, which were considered strong candidates, given that compounds of this class, such as neocarzinostatin and dynemycin, are well-known DNA-intercalating agents. However, gene deletion excluded both of these candidates, and LC-MS profiling did not detect any enediyene compounds in the strain extracts. Further analysis of the genome revealed another candidate, a type II polyketide synthase cluster (ST_PKS2), predicted to be responsible for the synthesis of a glycosylated aromatic polyketide potentially related to another class of DNA-intercalating agents, including daunomycin. Disruption of the ST_PKS2 cluster led to disappearance of the DNA-interfering activity. In order to identify and characterize the bioactive compound in a fast and accurate way, Kersten *et al*. performed differential metabolomics on the wild type and the disruption mutant. Several consistent differences were found between the metabolomic profiles of both strains in a mass spectrometric analysis. To identify which of the peaks that were present in the wild type profile but not in the disruption mutant corresponded to the DNA-interfering molecule, tandem mass spectrometry analysis was performed on all the differentiating peaks. Desoxysugar fragments were detected in the fragmentation pattern of one of the molecules, indicating that it is a glycosylated compound. By NMR analysis, the unknown compound was identified as lomaiviticin C, which is a glycosylated aromatic polyketide [24] known to possess DNA-interfering activity. Thus, the use of metabolomics allowed the rapid identification of the molecule responsible for the observed bioactivity as a known compound.

Further acceleration of the dereplication by metabolomics requires faster automated identification of detected metabolites in a profile. Cuthbertson *et al*. [25] described the development of an accurate mass—time tag system for rapidly identifying plant natural products by creating a database which contains the accurate mass, retention time and MS/MS fragmentation patterns of known compounds, including many secondary metabolites. A complementary approach using multistage mass spectrometry (MS^n^) was introduced recently by Rojas-Cherto *et al*. [26]. Their method can not only be used to identify already known compounds present in the generated database, but also allows to subtract substructures from spectra of unidentified compounds, allowing to focus on (or away from) particular classes of metabolites [26].

In a more ambitious experimental design, Krug *et al*. [27] applied metabolomics methodology to characterize the intra-species diversity of the secondary metabolome of 98 *Myxococcus xanthus* strains from 78 different locations. They used a UPLC-coupled high-resolution ESI-TOF mass spectrometry setup to analyse their samples. Several compounds already know to be present in *M. xanthus* were found in all or large subsets of the analyzed strains. More surprisingly, an additional 37 new candidate compounds were also detected in individual strains or subgroups of the species, revealing an unexpected intra-specific diversity of the secondary metabolome. This is an area of research that will benefit most directly from the high-throughput capacities of metabolomics, compared to genome-based approaches, as genome-sequencing at this level of taxonomic resolution will not only be inefficient but will also pose a major problem if the observed diversity is not due to differential absence/presence of gene clusters but the result of differential regulation of clusters that are shared throughout the species.

The high-throughput nature of metabolomics data acquisition is made particularly pertinent by the fact that the number of potential sources for new secondary metabolites is immense; almost all microbial organisms produce at least a dozen of these compounds, and most biological species have never been tested for their secondary metabolite profile. Traditionally, microbial strains for testing have been selected by their similarity to species with known potential for producing interesting compounds. For example, actinomycete bacteria have been intensely explored, due to the fact that many species are known to produce secondary metabolites with antibiotic activity [28]. Naturally, this leads to the problem that the same or very similar compounds tend to be detected repeatedly (as discussed above), and the increasing challenge of dereplication was one of the reasons for a declining interest in secondary metabolites as leads for drug discovery [29,30]. Genome mining followed by the “awakening” of cryptic secondary metabolites is a recent complementary approach [9,31,32], but it is still costly and time consuming and rarely performed on a large scale. Metabolomics is the obvious alternative (or complement) to these strategies [33].

By the high-throughput analysis of the metabolome profiles of microbial strains, organisms can be clustered according to the metabolites that they produce. This is well illustrated in a recent study by Hou *et al*. [33], directed at diverse microbes from unusual niches, including marine *Streptomyces* species cultivated from tropical ascidians. They establish a general strategy for using metabolomics to prioritize microbial strains for more detailed drug discovery efforts based on their metabolomic profiles. They exploit this strategy to complement the existing genome-based and taxonomy-based approaches, showing that phylogenetically close strains (based on 16S RNA analysis) are not necessarily producing the same secondary metabolites. They cluster strains according to similarities in metabolite profile using principal component analysis and identify compounds that are unique to individual strains (or groups of strains) based on the loading plots. Using the accurate mass information as a guide they followed up a selection of these unique compounds and discovered, e.g., a new bottromycin A2 analogue, bottromycin D, which is active against methicillin-resistant *Staphylococcus aureus* (MRSA), which was then confirmed by genome sequencing to identify the biosynthetic pathway [34]. 

Advanced mass spectrometry methods have recently begun to make an impact on secondary metabolite discovery. It has been known for some time that a microbial strain containing cryptic secondary metabolite clusters can produce novel compounds not only if these clusters are activated by genetic manipulation or different growth media, but also when production is forced by co-cultivation with other strains (see, e.g., [35]). It is now possible to measure such interactions using imaging mass spectrometry, which enables the determination and visualization of the spatial distribution of metabolites across microbial colonies. For example, Yang *et al*. [36] applied MALDI-TOF-imaging mass spectrometry to characterize the metabolic interactions between *Bacillus subtilis* and *Streptomyces coelicolor* growing in close physical neighbourhood on agar plates. They characterize the spatial distribution and chemical identity of compounds produced by individual and interacting colonies [37]. Initially the method was limited to measuring metabolites on the surface of growing cultures, neglecting the potentially more important compounds diffusing throughout the agar medium [38]. Watrous *et al*. [38] overcome this limitation and create complete 3D models of metabolite distribution from MALDI-TOF-IMS data, including profiles deep inside the agar, allowing unprecedented insight into the possible function of unknown compounds. This will enhance our search strategies for novel bioactive secondary metabolites, which are very often functioning in species–species interactions in their natural context.

The advanced mass spectrometry toolkit for microbial metabolomics is complemented by another strategy proposed by the same group to monitor living microbial populations using nano-spray desorption electrospray ionization (nanoDESI) mass spectrometry [39]. Samples can be analysed directly from living cultures on agar plates and visualized to identify the dynamics of the molecular interface of microbial co-cultures. By analyzing tandem mass spectrometry fragmentation data and identifying shared substructures between metabolites it is possible to detect and visualize entire families of related compounds across time and between different microbial species on a massive scale [39].

## 3. Metabolomics for Secondary Metabolite Production

Metabolomics approaches to the study of secondary metabolites have seen a recent boost due to the advent of Synthetic Biology. In this field, metabolomics not only serves the purpose of analyzing individual compounds on a large scale, but is particularly important as a generic debugging tool for engineered microbial production systems.

Synthetic Biology of secondary metabolite production comes in different flavours; it can be restricted to the awakening of the cryptic metabolite production encoded in newly sequenced genomes, but it can also involve the generation of novel chemistry by modifying and recombining the modular biosynthesis apparatus found in nature, as well as more ambitious re-engineering strategies, e.g., replacing the native regulatory machinery by fine-tuned designer systems (“refactoring”) or the improvement of production levels, e.g., by redeployment of primary metabolism [40]. In all of these cases, not only the synthesis of the bioactive compounds of interest will change, but there will also be off-target effects on metabolism in general.

Such widespread effects on the metabolic profile have been demonstrated, e.g., in a careful metabolomics study of the consequences of the overexpression of an ncRNA-based regulatory element in *Streptomyces coelicolor* [41]. The ncRNA was supposed to target a single enzyme involved in nitrogen fixation (glutamine synthetase, GSI), but in addition to effects on nearby metabolites, such as glutamine and glutamate, its induction also led to pervasive and rapid changes in metabolite levels throughout the metabolic network of the organism. An exceptional level of biological and technical replication of the metabolite profiling experiments was required to ensure that the observed metabolite dynamics were robustly reproducible.

Other applications of metabolomics in the debugging of engineered microbes are the identification of metabolic bottlenecks, such as the accumulation of unwanted or toxic side products and intermediates, or the depletion of required precursors. In these cases, metabolomics provides an unbiased overview of the metabolic status of a system and its changes due to the overproduction of compounds of interest, which in combination with systems biological modelling can drive cycles of refined engineering [42]. For example, Cheng *et al*. [43] characterize the metabolome of *Streptomyces lydicus* in different culture conditions and identify critical precursor compounds of streptolydigin (proline and glutamate) by their depletion in overproducing cultures. Subsequently, they could show that addition of exogenous glutamic acid and proline lead to a dramatic increased in the production of streptolydigin, not affecting the growth of the strain. Here the metabolomics approach resulted in a straightforward identification of a major bottleneck in the system, without the need for a detailed understanding of the underlying metabolic and regulatory network.

The unbiased nature of metabolomics is particular important in this context, as the true metabolic capacity of many organisms is only incompletely known, as can be demonstrated, e.g., by ^13^C-based metabolomics studies, which reveal the rapid distribution of labelled precursor metabolites through unexpected areas of the metabolome [44,45,46]. This kind of labelling approach can also be applied to discovering novelty in secondary metabolism: while the technique usually requires a single source of carbon or nitrogen, in context of secondary metabolite discovery this can be achieved by tracing the distribution of a class-specific precursor to side branches of the metabolic network [14].

## 4. Conclusions

High-throughput unbiased metabolite profiling has only begun to make its impact on the study of secondary metabolism. Recent advances in technologies (including high-resolution mass spectrometry and metabolite imaging mass spectrometry) expand the scope of metabolomics beyond the detection of well-characterized compounds in highly controlled settings. It offers new opportunities to access unexplored ecological niches with unusual metabolite diversity and to understand the function and dynamics of metabolite production, both in the natural environment and in the engineered production strains of Synthetic Biology.
